# A polymer that reinforces luminal barrier function and attenuates inflammation in murine colitis

**DOI:** 10.21203/rs.3.rs-8290892/v1

**Published:** 2026-06-19

**Authors:** Luiz Fernando Wurdig Roesch, Jessica Xhumari, Ariana Tamura, Amanda Ojeda, Oluwamayowa Akinsuyi, Diego Pedro, Christian Jobin, Ye Yang, Franccesco Boeno, Orlando Laitano, Marta Gomez, Jorge Frias-Lopez, Wallace Sawyer, Brent Sumerlin, Borhane Ziani, Madeline Gish, Ramon Sun

**Affiliations:** University of Florida; University of Florida; University of Florida; University of Florida; University of Florida; Moffitt Cancer Center; UNC School of Medicine; University of Florida; University of Florida; University of Florida; University of Florida; University of Florida; Moffitt Cancer Center; University of Florida; University of Florida; University of Florida; University of Florida

## Abstract

Wet epithelial surfaces are protected by mucus, a hydrated gel formed primarily by heavily glycosylated mucins that supports lubrication and limits microbial access to the epithelium. In the colon, barrier function depends on mucus properties that restrict bacterial penetration. Disruption of the mucus barrier integrity allows bacteria and bacterial toxins to contact the epithelium, promoting inflammation and colitis. When sustained, barrier dysfunction and inflammation are associated with an increased risk of colorectal cancer. Although current therapies primarily target downstream immune responses, there remains interest in approaches that directly reinforce mucosal barrier function. Here, we tested the hypothesis that reinforcing luminal separation between bacteria and the epithelium reduces microbial product sensing and innate inflammatory activation in colitis. We developed a polymer that reinforces luminal barrier function and characterized its therapeutic potential in two murine models of compromised epithelial-microbial segregation: a genetic model with impaired mucus production (Agr2-knockout) and a chemically induced injury model (dextran sulfate sodium colitis). Oral administration of the polymer was associated with improved disease indices, reduced histopathological injury, and broad suppression of innate inflammatory programs. Multi-omics profiling further indicates attenuation of LPS-associated signaling and enhancement of epithelial homeostatic signatures following treatment. Our results suggest that reinforcing luminal barrier function may represent a therapeutic approach for attenuating intestinal inflammation by limiting host exposure to luminal microbial products.

## INTRODUCTION

The mucus layer of the gut mucosal barrier serves as the frontline defense against microbial insults from the gut^1^. It creates a physicochemical barrier between microbes and intestinal tissues while still allowing nutrients, water, ions, and gases to diffuse^2^. When intact, it acts as a selective physicochemical sieve, reducing immune activation. When the integrity of this barrier is compromised, as seen in various gastrointestinal conditions, including inflammatory diseases, colorectal cancer, and numerous enteric infections^3,4^, bacteria and toxins translocate from the intestinal lumen into the underlying tissues, triggering sustained activation of pro-inflammatory cytokines, inducing dysregulation of paracellular tight junctions, and chronic immune activation^5,6^. Chronic inflammation is frequently accompanied by alterations in microbial community composition and abundance, including increased abundance of facultative anaerobes and loss of obligate anaerobes, contributing to dysbiosis^7–9^.

Because barrier dysfunction plays a central role in intestinal inflammation, there is growing interest in experimental approaches that maintain epithelial-microbial spatial segregation without directly suppressing immune pathways^10,11^. Prior efforts have explored dietary and microbial strategies to modulate the luminal environment and epithelial interface, with varying degrees of success^12,13^. However, the extent to which non-immunosuppressive interventions can modulate inflammatory outcomes in the setting of mucus barrier dysfunction remains incompletely understood. Experimental systems that allow direct in vivo testing of barrier-directed interventions are therefore needed to clarify how modifying the epithelial-luminal interface influences inflammation and tissue homeostasis.

In this study, we developed a polymer capable of reinforcing luminal barrier integrity and characterized its therapeutic potential to attenuate inflammation in colitis. The polymer is synthesized via random copolymerization of *N,N*-dimethylacrylamide^14,15^, a biocompatible and hydrophilic polymer with a non-peptide backbone commonly used in drug delivery and tissue engineering^16^. Additionally, the polymer incorporates phenylboronic acids moieties, which have previously been shown to bind cis-diol-containing biomolecules^17–19^. Because bacterial glycoconjugates and host glycoproteins contain cis-diol-rich carbohydrate structures, these interactions may contribute to luminal retention and barrier-associated properties. Here, we examined the effect of the polymer in two complementary murine models of mucus barrier dysfunction: *Agr2*-knockout (*Agr2*^−/−^) mice, which exhibit impaired mucus production, and dextran sulfate sodium (DSS)- induced colitis, a model of acute disruption of the mucus and epithelial barrier. Across both preclinical models, we evaluated clinical disease indices, histopathology, inflammatory signaling, and microbiome composition. Our results indicate that polymer administration is consistently associated with reduced inflammation and improved tissue pathology, consistent with improved epithelial-microbial spatial segregation and reduced exposure to luminal bacteria and PAMPs.

## RESULTS

### Polymer Physicochemical Properties

**Figure 1:**
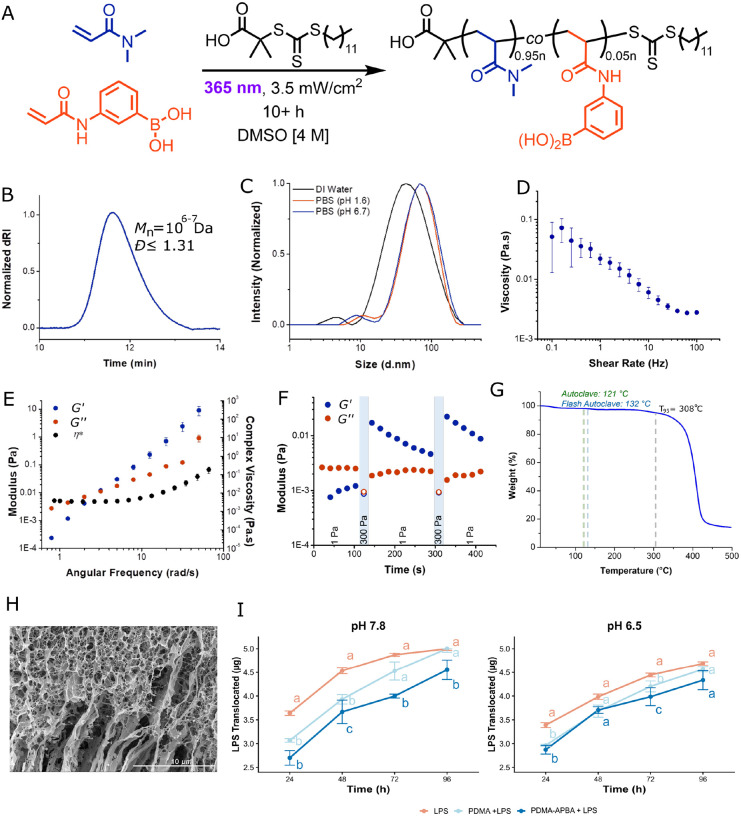
Polymer physicochemical characterization and bacterial lipopolysaccharide retention. **A.** DMA and APBA monomers were polymerized in DMSO under UV irradiation (365 nm) using DDMAT as the chain-transfer agent, yielding a predominantly DMA-based polymer with ~5% APBA incorporation. **B.** Size exclusion chromatography (SEC) demonstrating the formation of a high-molecular-weight polymer with a relatively narrow molecular weight distribution (*Đ* ≤ 1.31), indicated by the presence of a single dominant elution peak. Molecular weight distributions were calculated using the Wyatt ASTRA software. **C**. Hydrodynamic diameter (*D*_h_) of the polymer (5 mg/mL) in different pH conditions as determined by dynamic light scattering (DLS). **D.** Polymer viscosity (η) as determined through linear shear rheology. Steady shear rheology showed a progressive decrease in viscosity with increasing shear rate, consistent with shear-thinning behavior. **E.** Oscillatory frequency sweep showing the storage (*G′*) modulus, loss (*G″*) modulus, and complex viscosity (η*) as a function of angular frequency. Both viscoelastic moduli increased with frequency, with *G′* exceeding *G″* at higher frequencies, indicating frequency-dependent viscoelastic behavior. **F.** Stress sweep rheology showing a reversible gel-sol transition under elevated mechanical stress. **G.** Thermal stability as determined by thermal gravimetric analysis (TGA). **H.** Cryogenic scanning electron microscopy of the hydrated polymer. **I.** Transwell diffusion assay evaluating lipopolysaccharide (LPS) translocation across polymer barriers at pH 7.8 and pH 6.5. Cumulative FITC-LPS transport to the basal chamber was measured over time following exposure to PDMA alone or PDMA-APBA polymer formulations. Data represent mean ± SEM. Different letters indicate statistically significant differences between groups at each time point based on Tukey’s post hoc test (p < 0.05).

The polymer was synthesized via photoiniferter methods to yield ultrahigh molecular weight polymers (> 1 MDa) with low dispersity^20,21^. The composition consists of hydrophilic *N,N*-dimethylacrylamide (DMA) repeat units and incorporates 6 ± 1.21% of 3-acrylamidophenylboronic acid (APBA) to exploit its capability to form reversible covalent complexes with 1,2- or 1,3-*cis* diols^22^ (Figs. 1A and Figure S1). The polymerization strategy offers a one-pot synthetic method performed at ambient pressure and temperature, and it is scalable to multigram reactions without specialized glassware or equipment. Size exclusion chromatography (SEC) (Fig. 1B) indicates consistent and reproducible polymerization across batches, with *M*_n_ of 1.80 ± 0.032 MDa and narrow dispersity values (*Đ* = 1.16 ± 0.085) (Supplementary Table S1). For tracking, the polymer was fluorescently tagged with fluorescein *O*-methacrylate (FMA), which was directly incorporated into the polymer backbone during polymerization. (Supplementary Table S1).

Dynamic light scattering showed that the polymer maintained a comparable hydrodynamic diameter (~ 75–81 nm) across pH conditions spanning the gastrointestinal tract (Fig. 1C). To probe its macromolecular behavior under physiologically relevant flow, viscosity (η) was measured across shear rates that approximate gastrointestinal transit. The polymer demonstrated decreasing viscosities with increasing shear rates (γ), which indicates shear-thinning behavior under steady shear conditions (Fig. 1D).

Shear stress-dependent experiments showed the storage modulus (*G′*) exceeds the loss modulus (G″) at higher angular frequencies, indicating frequency-dependent viscoelastic behavior consistent with transient network formation (Fig. 1E). The increase in complex viscosity (η*) with frequency supports the presence of reversible intermolecular associations that contribute to structural cohesion under dynamic conditions. Stress sweep experiments demonstrated that the polymer undergoes a reversible transition between viscous- and elastic-dominated regimes as a function of applied stress (Fig. 1F). At low stress (1 Pa, 0–120 s), the material displayed predominantly viscous behavior (*G″* > *G′*). At intermediate stresses, the elastic modulus (*G′*) increased relative to *G″*, indicating formation of transient intermolecular associations characteristic of a weak, dynamically structured network. At high stress (300 Pa), the elastic modulus decreased substantially, indicating disruption of reversible intermolecular interactions and recovery of solution-like behavior. These findings are consistent with a physically crosslinked polymer system capable of dynamic restructuring in response to mechanical forces.

Thermal gravimetric analysis was performed to confirm that the polymer backbone provides sufficient thermal stability to withstand common sterilization procedures without structural degradation (Fig. 1G). The high decomposition temperature (T_95_ = 308°C) indicates that the polymer maintains chemical integrity under conditions well exceeding those encountered during autoclaving (121–132°C) or storage, supporting its practical applicability as a biomedical material.

Cryogenic scanning electron microscopy revealed a heterogeneous, interconnected nanoscale porous polymer network spanning multiple length scales characteristic of hydrated polymer architectures (Fig. 1H). The material exhibited dense fibrillar domains interconnected by nanoscale porous compartments, suggesting polymer entanglement and spatial heterogeneity within the hydrated matrix.

Having established the polymer physicochemical properties, we next asked whether these properties translate into functional barrier behavior, and to what extent the boronic acid chemistry contributes beyond the bulk polymer matrix. We selected bacterial lipopolysaccharide (LPS) as the probe: it bears cis-diol motifs that can engage APBA through reversible boronate ester formation, and its translocation across the epithelium is a principal driver of the inflammatory cascades examined in later sections. To delineate the relative contributions of chemical interactions and the nonspecific physicochemical effects of the polymer, we assessed LPS translocation across different polymer compositions under two pH conditions: The hydrophilic PDMA backbone alone, without APBA incorporation, and the original copolymer formulation with DMA and APBA incorporation (Fig. 1I). Both polymers reduced LPS translocation at early time points, indicating that the polymer matrix itself provides a physical barrier to LPS transport. However, this effect diminished over time in the PDMA-only formulation, with LPS translocation approaching that of the control (no-polymer) condition by 96h. In contrast, the PDMA-APBA formulation sustained reduced LPS translocation (Fig. 1I). These results indicate that while nonspecific physicochemical properties of the polymer contribute to initial barrier effects, incorporation of APBA provides an additional mechanism that provides LPS retention. Phenylboronic acids typically exhibit pK_a_ values around 8.5–9.0^23^, meaning that boronic acid-diol complexation is expected to weaken at lower pH. LPS retention by the APBA-containing polymer was also lower at pH 6.5 than at pH 7.5, the directionality expected for boronate-ester complexation and consistent with an APBA-mediated contribution to retention.

### In-vivo tolerability, spatial localization, and gastrointestinal transit

**Figure 2:**
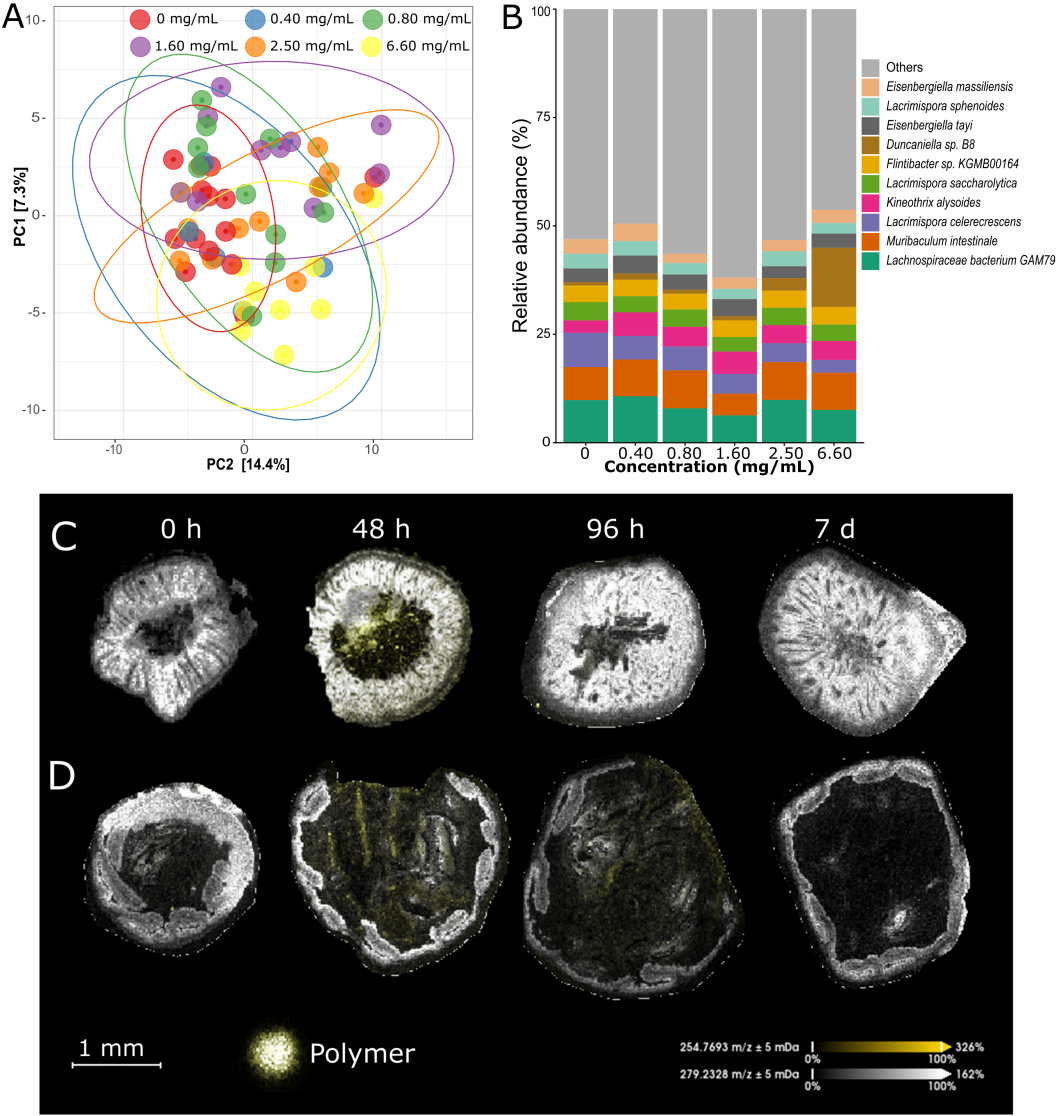
**A**. Principal coordinates analysis (PCoA) based on Aitchison distances of fecal microbial community composition in wild-type mice treated with increasing polymer doses. Distances between samples reflect differences in community structure (beta diversity). Ellipses represent 95% confidence intervals around group centroids. Axis labels indicate the percentage of variance explained by each principal coordinate. **B.**Relative abundance of the 10 most abundant bacterial species identified in fecal samples across polymer treatment groups. Data were obtained from wild-type mice treated with increasing concentrations of polymer. **C-D**. Representative MALDI mass spectrometry imaging (MALDI-MSI) of duodenal (C) and colonic (D) sections collected from wild-type mice following polymer administration. Mice received polymer in drinking water for 2 d, after which treatment was discontinued. Tissues were collected immediately after treatment cessation (control), or at 48 h, 96 h, and 7 d after polymer withdrawal. Polymer-associated ion signals (yellow) are shown overlaid on tissue morphology (grayscale).

We investigated the *in-vivo* short-term tolerability, dosing parameters, and gastrointestinal transit of the polymer to establish its physiological compatibility and distribution in adult wild-type C57BL/6J mice for ten days. For the purposes of this experiment, we defined tolerability as the absence of evident adverse clinical effects across the range of administered doses during short-term exposure, rather than pharmacological tolerance or adaptation. Comprehensive safety assessment, pharmacokinetic characterization, and long-term exposure studies remain important areas for future work. Endpoints included clinical symptoms (dysphagia, consistency of feces, intestinal bleeding), body weight, rectal prolapses, gut microbiome changes, and mortality. The polymer was provided in drinking water to ensure constant exposure while minimizing stress from repeated gavage. Daily water consumption was not statistically affected across groups, averaging 5.7 ± 1.3 mL/d in polymer-treated mice and 6.0 ± 2.0 mL/d in the control group. No adverse clinical effects were observed at any dose, and the survival rate was 100%. Figure S2 shows the effect of different treatment doses on the body weight of wild-type mice over time. Using linear mixed modeling, we observed a slight, expected increase in body weight over time in all treatments and controls (β = 0.66, SE = 0.29, p-value = 0.024), consistent with normal growth in young adult mice (Supplementary Table S2). None of the polymer doses significantly changed the mice body weight compared to controls (p-value > 0.05). A microbiome analysis was conducted to monitor alterations in bacterial community composition and diversity driven by treatment. No significant changes in alpha diversity were observed in the treated wild-type mice or untreated controls, irrespective of the dose used (Figure S3). Minor changes in beta diversity (Fig. 2A, Supplementary Table S3) and species composition (Fig. 2B, Supplementary Table S4) were observed with the highest polymer dose (6.60 mg per mL, approximately 40 mg per day). Differential abundance analysis indicated that those changes were associated with variations in the relative abundance of taxa within the order *Clostridiales*. Other doses tested did not cause detectable changes in the microbial abundance (Supplementary Table S4).

To assess gastrointestinal transit, the polymer was fluorescently labeled with fluorescein methacrylate. Fecal pellets were collected every other day from day zero to ten, and their fluorescence was measured. The results show that fecal fluorescence increased with the dose, consistent with dose-dependent gastrointestinal transit and fecal recovery of the polymer. (Figure S4A). A fecal fluorescence signal was observed within 24 hours of treatment initiation. Upon cessation of dosing, fecal fluorescence decreased over time and approached baseline levels, consistent with reduced fecal output of fluorescent material after exposure ended. (Figure S4B). No adverse clinical effects were observed after discontinuation of the treatment.

Although the polymer ultrahigh molecular weight (> 1 MDa) precludes intact-chain detection by conventional MALDI, label-free MALDI-MSI resolves polymer location through low-mass fragment signatures, providing spatial information without requiring fluorescent labels that could alter transit. MALDI imaging of duodenal and colonic sections collected at the end of the two-day polymer treatment period (48 h from experiment initiation) showed predominant localization of the polymer within the lumen (Fig. 2C, D). Polymer signal decreased progressively following treatment cessation. At 96 h (48 h after treatment cessation), no polymer signal was detected in duodenal sections, whereas colonic sections still exhibited detectable polymer retention, suggesting prolonged persistence in the colon. No polymer signal was detected in either tissue at 7 d (5 d after treatment cessation). The prolonged colonic signal suggests region-dependent retention, potentially influenced by slower distal transit and local luminal conditions, including pH-dependent boronic acid interactions. However, this mechanism was not directly tested in vivo.

#### Polymeric mucus surrogate preserves epithelial-microbial spatial segregation and attenuates inflammation in mucus-deficient Agr2^−/−^ mice

**Figure 3:**
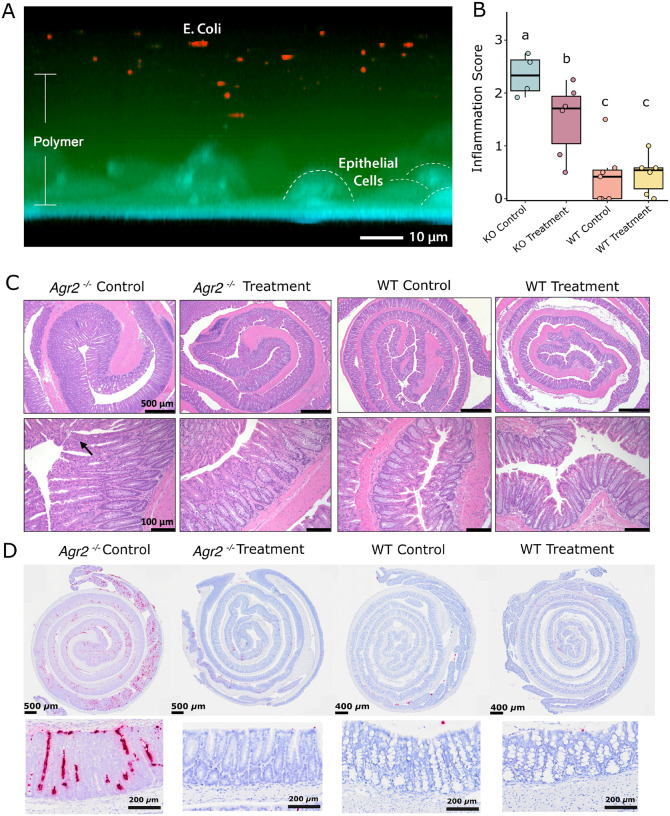
Polymer treatment preserves epithelial-microbial spatial separation and attenuates intestinal inflammation in mucus-deficient *Agr2*^−/−^ mice. **A.** Three-dimensional confocal microscopy image showing in vitro spatial separation between *E. coli* (red) and epithelial cells (cyan) in the presence of the polymer layer (green). **B**. Histopathological inflammation scores of colonic tissues from *Agr2*^−/−^ and WT mice following polymer treatment. Polymer-treated *Agr2*^−/−^ mice exhibited reduced inflammation compared to untreated *Agr2*^−/−^ controls, whereas no significant differences were observed between WT groups. Groups sharing the same letter are not significantly different (p < 0.05). **C**. Representative hematoxylin and eosin-stained colonic Swiss-roll sections showing reduced inflammatory infiltration, epithelial hyperplasia, and tissue disruption in polymer-treated *Agr2*^−/−^ mice relative to untreated *Agr2*^−/−^ controls. WT mice showed minimal histopathological alterations following treatment. The arrow indicates inflammatory leukocyte infiltration. **D**. Representative RNA in situ hybridization (RNAscope) images targeting bacterial 16S rRNA in colonic Swiss-roll sections. Untreated *Agr2*^−/−^ mice exhibited increased bacterial penetration into the epithelium, whereas polymer-treated *Agr2*^−/−^ mice and WT controls showed minimal bacterial signal within intestinal tissues.

The gastrointestinal mucus layer normally limits direct contact between microbes and the epithelium, attenuating inflammatory activation. We tested whether the polymer could provide a comparable physical barrier in vitro. Using a controlled epithelial-surface imaging assay, we observed that polymer treatment increased the spatial separation between bacteria and adherent epithelial cells (Fig. 3A). Because the hTCEpi cells used in this assay are not intestinal epithelial cells, the results were interpreted only as evidence of physical separation, not as a model of colonocyte-specific epithelial responses.

After confirming that the polymer promotes epithelial-microbial spatial segregation in vitro and assessing its short-term tolerability, dosing regimen, and gastrointestinal transit in WT mice, we evaluated its biological effects in a murine model of primary mucus deficiency. This mouse model is predisposed to colitis due to the knockout of anterior gradient 2 (*Agr2*)^24^. The lack of MUC2 was confirmed through proteomic analysis of colonic tissue (Figure S5). The polymer was administered daily via drinking water at 6.60 mg/mL for 17 d to *Agr2*^−/−^ mice and WT littermate controls, and each group was compared with its respective untreated control (n = 7–9 per treatment). Clinical indices, histopathology, microbiota composition, and proteomic profiles of colon and spleen were assessed to characterize the inflammatory and systemic responses. Colonic and splenic proteomes were interrogated for enrichment in pathways associated with cellular responses to toxic stimuli, including detoxification, stress response, and xenobiotic metabolism, in *Agr2*^−/−^ and WT mice after 17 d of treatment.

Colonic histopathology was evaluated to assess associated tissue-level inflammatory outcomes as downstream effects of reduced epithelial exposure to luminal microbial products. Untreated *Agr2*^−/−^ mice displayed higher inflammation scores characterized by epithelial hyperplasia, leukocyte infiltration, and crypt distortion (score = 2.33 ± 0.40; Fig. 3B and C). In contrast, polymer-treated *Agr2*^−/−^ mice exhibited significantly lower histological scores (1.50 ± 0.69), whereas WT mice showed no difference between treated and untreated groups (WT control = 0.43 ± 0.54; WT treatment = 0.46 ± 0.37).

The spatial relationships between the epithelium and bacteria in vivo were observed using RNA in situ hybridization targeting bacterial 16S rRNA in colonic tissue sections. Untreated *Agr2*^−/−^ mice exhibited extensive epithelial-associated bacterial signal across the colon, whereas polymer-treated *Agr2*^−/−^ mice, as well as WT controls, showed reduced epithelial-associated bacteria and limited crypt invasion (Fig. 3D).

**Figure 4:**
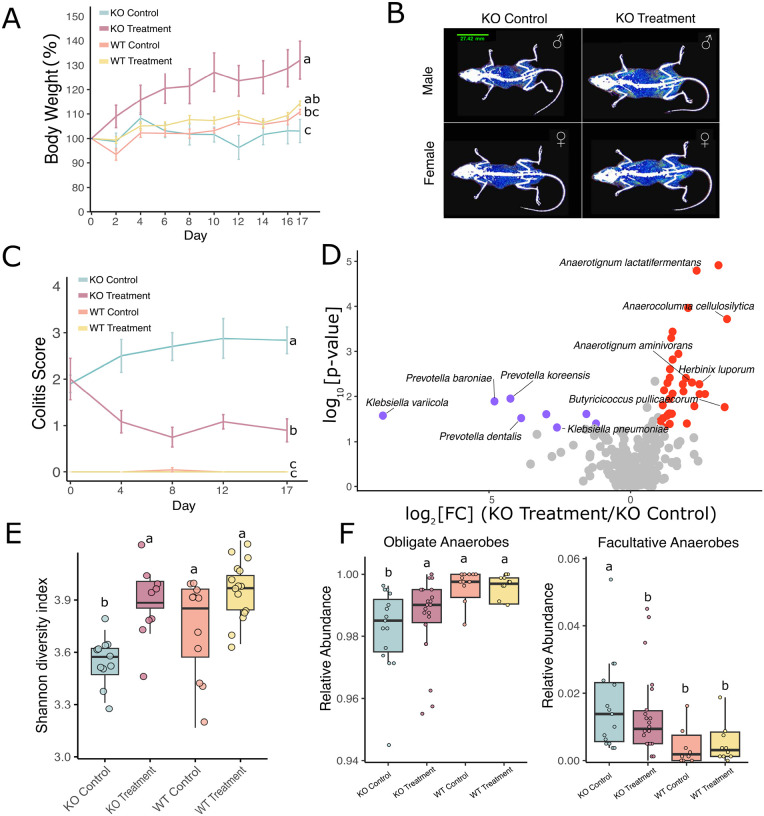
Polymer treatment improves body weight, colitis scores, and microbiome composition in *Agr2*^−/−^ mice. **A.** Body weight progression in *Agr2*^−/−^ and WT mice during polymer treatment. Polymer-treated *Agr2*^−/−^ mice exhibited progressive body weight gain over 17 d compared to untreated *Agr2*^−/−^ controls, whereas WT mice showed minimal treatment-associated changes. Groups sharing the same letter at day 17 are not significantly different (p < 0.05). **B**. Representative postmortem DEXA body scans of male and female *Agr2*^−/−^ control and polymer-treated mice showing increased body mass following polymer treatment. **C**. Longitudinal colitis scores in *Agr2*^−/−^ and WT mice during polymer treatment. Polymer-treated *Agr2*^−/−^ mice exhibited reduced colitis severity beginning at day 4 relative to untreated *Agr2*^−/−^ controls, whereas WT groups maintained scores near zero throughout the experiment. Groups sharing the same letter are not significantly different (p < 0.05). **D.** Volcano plot showing the differentially abundant bacterial species identified between polymer-treated and untreated *Agr2*^−/−^ mice over the 17-day treatment period. Species increased in polymer-treated *Agr2*^−/−^ mice are shown in red, whereas decreased species are shown in blue. **E**. Shannon diversity index of fecal microbial communities in *Agr2*^−/−^ and WT mice following polymer treatment. Polymer-treated *Agr2*^−/−^ mice exhibited increased microbial diversity relative to untreated *Agr2*^−/−^ controls, whereas WT mice showed no significant treatment-associated differences. Groups sharing the same letter are not significantly different (p < 0.05). **F**. Relative abundance of obligate and facultative anaerobes in fecal microbial communities. Polymer treatment in *Agr2*^−/−^ mice increased the relative abundance of obligate anaerobes and reduced facultative anaerobes toward levels observed in WT mice. Groups sharing the same letter are not significantly different (p < 0.05).

The body weight of treated *Agr2*^−/−^ mice increased compared to the untreated *Agr2*^−/−^, while remaining unaffected in WT controls (Fig. 4A and B). The weight gain had a large effect size (β = 11.77, SE = 2.13, p-value < 0.0001, Cohen’s *d* = 0.86). By the end of the experiment, the average weight of untreated *Agr2*^−/−^ mice was 14.2 ± 1.4 g, approximately 35% lower than that of WT mice of the same age. Notably, *Agr2*^−/−^ mice are susceptible to colitis and consequently weigh less than their WT counterparts. Post treatment, *Agr2*^−/−^ mice reached an average weight of 20.5 ± 1.8 g, comparable to the weight of WT mice (22.2 ± 3.2 g untreated; 24.0 ± 3 g treated). Body weight improvement (36.3%) was observed only in the treated *Agr2*^−/−^ mice. Using differences in weight and clinical scores as indicators, we finalized the experiment on day 17 to minimize pain in the control group, as the treated mice had reached a body weight similar to that of the WT.

A composite colitis score, which integrated weight loss, diarrhea, and blood in feces, was also measured in *Agr2*^−/−^ mice (Fig. 4C). After 4 d, a 45.8% reduction from day 0 was observed in the treated group with a large effect size (β = 1.79, SE = 0.43, p-value = 0.002, Cohen’s *d* = 2.09). The partial eta squared (η_p_^2^) for the effect of treatment on colitis score was 0.56, indicating that the treatment explained 56% of the variance. The colitis score differences remained significant until the end of the experiment (day 17, β = 1.93, SE = 0.45, p-value = 0.001, Cohen’s *d* = 2.86), a 68.2% difference compared with untreated *Agr2*^−/−^ controls.

The microbiome analysis, based on full-length 16S rRNA gene sequencing, identified 393 bacterial species from 72 fecal samples collected during the experiment. The differentially abundant species between treated vs. untreated *Agr2*^−/−^ mice at the end of the experiment are shown in Fig. 4D, with the top 10 species labeled. When using a fold change cutoff of 2 and an adjusted p-value of 0.05, 52 species were significantly changed (Supplementary Table S5). Among the 39 species that significantly increased with treatment were *Anaerotignum lactatifermentans, Anaerotignum aminivorans, Anaerocolumna cellulosilytica, Herbinix luporum*, and *Butyricicoccus pullicaecorum*, species associated with fiber fermentation and short-chain fatty acid production. In contrast, 13 species decreased after treatment, including opportunistic pathogens (*Klebsiella pneumoniae, Klebsiella variicola*) and *Prevotella* species that have been linked to mucosal inflammation. The Shannon diversity index, which is typically reduced during inflammation, increased in *Agr2*^−/−^ mice after treatment (Fig. 4E). Physiological hypoxia in the colonic epithelium was indirectly measured by the relative abundance of anaerobic, aerobic, and facultatively anaerobic microbes. To avoid daily variations commonly observed in the mouse microbiome^25,26^, we analyzed the samples by combining sequences from days 2, 8, 12, 16, and 17 of the experiment. The relative abundance of these microbes changed with treatment (Fig. 4F). Anaerobic microbes dominated the gut environment of the treated and untreated wild-type mice (Control = 0.992 ± 0.010; Treatment = 0.989 ± 0.015). The untreated *Agr2*^−/−^ mice exhibited disruption of anaerobiosis, characterized by a decrease in the relative abundance of anaerobic microbes (0.921 ± 0.114) compared with wild-type, and a corresponding increase in facultative anaerobes and aerobic microbes. After the treatment, an increase in the relative abundance of anaerobic microbes was observed (0.975 ± 0.044).

**Figure 5:**
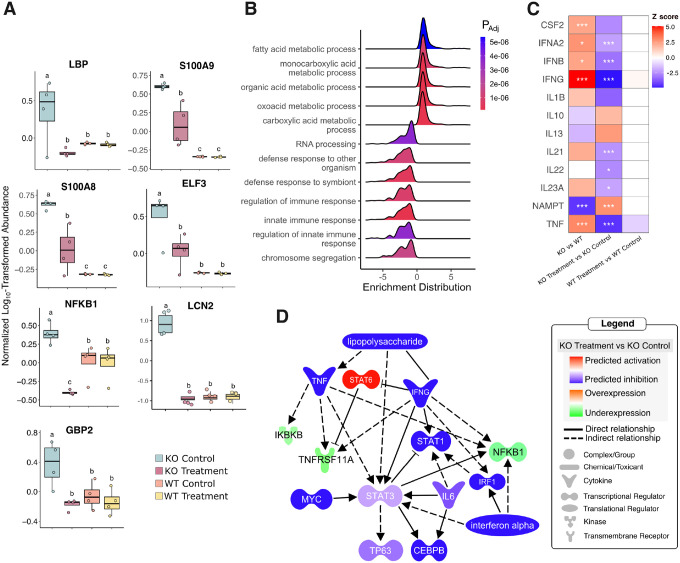
Polymer treatment suppresses inflammatory signaling pathways in *Agr2*^−/−^ mice. **A.** Normalized protein abundance of inflammatory and immune-response markers in colonic samples from *Agr2*^−/−^ and WT mice following polymer treatment. Polymer-treated *Agr2*^−/−^ mice exhibited reduced abundance of multiple inflammation-associated markers, relative to untreated *Agr2*^−/−^ controls. Groups sharing the same letter are not significantly different (p < 0.05). **B**. Gene set enrichment analysis (GSEA) of colonic proteomic profiles comparing polymer-treated and untreated *Agr2*^−/−^ mice. Pathways associated with metabolic processes, including fatty acid and carboxylic acid metabolism, were positively enriched following treatment, whereas pathways related to immune and defense responses were negatively enriched. **C**. Heatmap of predicted activation states for selected inflammatory upstream regulators identified by Ingenuity Pathway Analysis (IPA) of the colonic proteome. Colors represent activation Z-scores, with red indicating predicted activation and blue indicating predicted inhibition. Asterisks denote statistical significance (*p < 0.05, **p < 0.01, ***p < 0.001). **D**. IPA-derived network model highlighting predicted inflammatory regulatory interactions altered by polymer treatment in *Agr2*^−/−^ mice. Node color reflects predicted activation state based on activation Z-scores, with red indicating activation and blue indicating inhibition relative to untreated *Agr2*^−/−^ controls. Solid and dashed lines represent known direct and indirect molecular interactions, respectively.

Considering the observed changes in epithelial-microbial spatial segregation, the apparent reduction in luminal permeability, and shifts in anaerobic community composition, we next examined molecular signatures associated with inflammatory activation. We identified the molecular cascades underlying these physiological and microbial changes by profiling the distal colon proteome. We detected 6,028 proteins and identified 3,604 as significantly changed by treatment, with a False Discovery Rate (FDR) < 0.05. Consistent with reduced epithelial-associated bacterial signal and improved pathology, polymer treatment was associated with lower abundance of proteins linked to microbial product exposure and neutrophil-driven inflammation, including lipopolysaccharide-binding protein (LBP) with an Omega-squared (ω^2^) of 0.43, indicating a high effect size and FDR = 0.033, and lipocalin-2 (LCN2) with an Omega-squared (ω^2^) of 0.52, indicating a high effect size and FDR = 0.021, relative to untreated *Agr2*^−^/^−^controls (Fig. 5A). Additionally, we observed improvements in calprotectin dimers S100A8 (ω^2^ = 0.86, FDR = 3.43e-5) and S100A9 (ω^2^ = 0.88, FDR = 1.31e-5), interferon-inducible GBP2 (ω^2^ = 0.51, FDR = 0.016), epithelial regulator ELF3 (ω^2^ = 0.69, FDR = 0.002) and Nuclear Factor Kappa B Subunit 1(NFKB1) (ω^2^ = 0.69, FDR = 0.002) (Fig. 5A).

FITC-dextran translocation assays showed reduced recovery of fluorescent tracer in treated mice (Figure S6), suggesting decreased luminal permeability. However, because the polymer incorporates phenylboronic acids that can bind to cis-diol-containing biomolecules, we cannot fully exclude polymer-probe interactions that may influence FITC-dextran recovery. Therefore, FITC-dextran is interpreted here as an apparent translocation readout. The reduced luminal permeability is supported by proteomic evidence, including decreased abundance of LBP and LCN2 (Fig. 5A), established biomarkers of intestinal permeability.

To contextualize the protein-level changes within broader biological networks, we next performed Gene Set Enrichment Analysis (GSEA) on the colon proteome (Fig. 5B). This systems-level approach clustered proteins into annotated biological processes and revealed that treated *Agr2*^−/−^ mice showed significantly lower enrichment of pathways involved in immune activation and defense responses. Within the top 12 gene sets, we observed a negative enrichment in “regulation of innate immune response” with a Normalized Enrichment Score (NES) of −1.99 (P_Adj_ = 3.68E-06) as well as in “defense response to symbiont” (NES = −1.83, P_Adj_ = 4.66e-07). To further elucidate the upstream regulators of these immune-modulatory effects on the colon, we implemented Ingenuity Pathway Analysis (IPA), as shown in Fig. 5C. Upstream Regulator Analysis in IPA identified activation patterns of key cytokines, including tumor necrosis factor-α (TNF-α), interferon-γ (IFN-γ), and type I interferons (IFNA2, IFNB), along with inactivation of the protective nicotinamide phosphoribosyltransferase (NAMPT) in *Agr2*^−/−^ controls compared to healthy WT controls. Importantly, this dysregulation was shifted after treatment in *Agr2*^−/−^ mice (Z score ≥ 1.96, p < 0.05). Treated WT colon tissue displayed minimal changes in upstream cytokine regulators. Full upstream regulator statistics are provided in Supplementary Table S6. To visualize higher-order signaling cascades affected by treatment, we used IPA to generate a regulatory network comparing *Agr2*^−/−^ mice treated versus untreated *Agr2*^−/−^ controls (Fig. 5D). Upstream regulator analysis predicted LPS as an inhibited upstream driver, with downstream cytokines and transcription factors such as TNF and IL-6 also inhibited. This inhibitory cascade converged on STAT3, a transcriptional hub regulating inflammatory and proliferative responses. Moreover, regulators of cell proliferation and apoptosis, such as MYC, TP53, and CEBPB, were also suppressed, while STAT6 was activated, consistent with a broad attenuation of immune-activation pathways.

**Figure 6:**
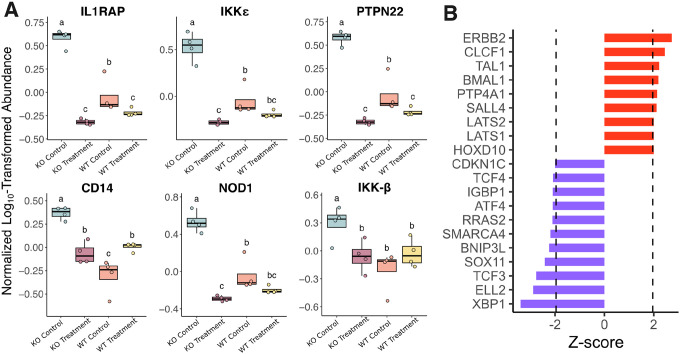
Polymer treatment modulates inflammatory-associated proteins and predicted regulatory signaling in the spleen of *Agr2*^−/−^ mice. **A**. Normalized abundance of inflammation-associated proteins in splenic proteomic profiles from *Agr2*^−/−^ and WT mice following polymer treatment. Polymer-treated *Agr2*^−/−^ mice exhibited reduced abundance of multiple inflammatory-associated proteins, relative to untreated *Agr2*^−/−^ controls, with several proteins shifting toward levels observed in WT mice. Groups sharing the same letter are not significantly different (p < 0.05). **B**. Top predicted upstream regulators identified by Ingenuity Pathway Analysis (IPA) based on differential splenic proteomic profiles between polymer-treated and untreated *Agr2*^−/−^ mice. Positive activation Z-scores (red) indicate predicted activation, whereas negative Z-scores (purple) indicate predicted inhibition of upstream regulators. Dashed lines indicate IPA activation Z-score thresholds for predicted regulator activation or inhibition.

Evaluation of the splenic proteome was performed to investigate the systemic consequences of immune modulation, a secondary effect of maintaining epithelial-microbial spatial segregation. A total of 6,059 proteins were detected in the spleen proteome, of which 3,455 showed significant changes in the treatment group (FDR < 0.05). Among the altered proteins were key mediators in pathogen recognition and inflammatory activation, which normalized toward WT levels after treatment in *Agr2*^−/−^ mice (Fig. 6A). Notably, the LPS receptor CD14 (ω^2^ = 0.80, FDR = 1.91e-4), the IκB kinases β (IKKβ) (ω^2^ = 0.45, FDR = 0.032) and IKKe (ω^2^ = 0.89, FDR = 1.34e-5), and the interleukin-1 receptor accessory protein IL1RAP (ω^2^ = 0.91, FDR = 4.94e-6). Additional key changes were observed in Nod1, a cytosolic sensor of bacterial peptidoglycan (ω^2^ = 0.90, FDR = 7.37e-6), and PTPN22, a phosphatase linked to autoimmunity and T-cell receptor signaling (ω^2^ = 0.91, FDR = 4.99e-6). Upstream Regulators were also analyzed using IPA to further understand how the polymer might drive changes in immune signaling in *Agr2*^−/−^ mice (Fig. 6B). In particular, the NF-κB inhibitor BMAL1 was significantly activated, while XBP1 and ATF4, which are involved in the unfolded protein response and NF-kB activation, were downregulated. While XBP1 is a central component of the adaptive unfolded protein response (UPR), in the context of *Agr2* deficiency, the predicted inhibition of XBP1 may reflect reduced inflammatory and secretory stress rather than impairment of UPR signaling. These observations in the spleen reveal a redirection of canonical NF-κB pathways in treated *Agr2*^−/−^ mice toward a healthy WT state.

To further evaluate whether the treatment posed systemic toxicological risks, we also analyzed the colon and spleen proteomes of *Agr2*^−/−^ and WT mice using GSEA to compare treated vs untreated mice after 17 d of treatment (Supplementary Tables S7-S10). In the colon and spleen of both *Agr2*^−/−^ and WT mice, no significant enrichment of any toxicological pathways, such as GO:0009636 (response to toxic substance) or GO:0097237 (cellular response to toxic substance), was observed. These findings show that the short-term treatment was not associated with systemic toxicological signatures. Overall, hydrophilic polymer treatment in *Agr2*^−/−^ mice was associated with broad attenuation of inflammatory features, as reflected by improvements in clinical symptoms, histopathology, microbiota composition, and proteomic profiles in the colon and spleen.

### Immunological outcomes in DSS-induced epithelial injury

Having observed attenuation of inflammatory outcomes as a treatment effect in a genetic model of mucus deficiency, we next evaluated the effect of the polymer on recovery from acute epithelial injury in the DSS-induced colitis model^27,28^. This model was used to determine whether the reduced epithelial exposure to luminal microbial products extended beyond genetically impaired mucus production to a mechanistically distinct context of chemically induced epithelial damage. Wild-type mice were administered 2.5% DSS (w/v) in drinking water for 4 d, followed by a 4-day recovery phase during which animals received either the polymer treatment or water alone.

**Figure 7:**
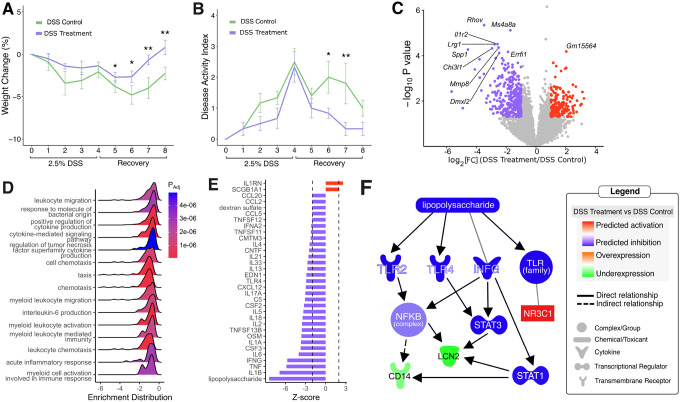
Polymer treatment accelerates recovery from DSS-induced colitis and suppresses inflammatory transcriptional programs. **A**. Body weight changes during DSS-induced colitis and recovery. Polymer-treated mice exhibited improved weight recovery after DSS withdrawal compared with untreated DSS controls. Asterisks indicate statistically significant differences between groups (*p < 0.05, **p < 0.01). **B**. Disease Activity Index (DAI) scores during DSS-induced colitis and recovery. Polymer-treated mice exhibited reduced disease severity during the recovery phase compared to DSS controls. Asterisks indicate statistically significant differences between groups (*p < 0.05, **p < 0.01). **C**. Volcano plot of differentially expressed genes identified by RNA-seq analysis of terminal whole-blood clots collected on day 8 from DSS-treated mice. Genes downregulated following polymer treatment are shown in purple, whereas upregulated genes are shown in red. **D**. Gene set enrichment analysis (GSEA) of blood transcriptomic profiles comparing polymer-treated and untreated DSS mice. Immune and inflammatory pathways, including leukocyte migration, cytokine-mediated signaling, and myeloid leukocyte activation, were negatively enriched following polymer treatment. **E**. Ingenuity Pathway Analysis (IPA) upstream regulator analysis predicting altered inflammatory signaling following polymer treatment in DSS-induced colitis. Negative activation Z-scores indicate predicted inhibition of inflammatory regulators, whereas positive Z-scores indicate predicted activation.

DSS exposure resulted in comparable weight loss across groups during the challenge phase, after which trajectories diverged during recovery (Fig. 7A). During recovery (days 4–8), percent weight change models showed a significant interaction between treatment and time (β = 0.693% per day, SE = 0.327, p = 0.040), indicating accelerated resolution in treated mice. By the end of recovery (day 8), the effect size of the weight change between treated and untreated mice was large (δ = −1.54, 95% CI [− 2.83, − 0.20]). These results indicate improved weight recovery during the post-DSS phase in polymer-treated mice. Furthermore, to track colitis symptoms such as diarrhea and blood in stool alongside weight changes, the Disease Activity Index (DAI) was recorded over time (Fig. 7B). During the DSS challenge, the DAI rose comparably in both groups, and by day 4, all the mice reached peak scores at 2.4, indicating moderate DSS injury. Pairwise contrasts within the mixed-effects model showed faster symptom resolution in treated mice during the recovery phase. At day 6, controls maintained higher DAI scores (1.62 ± 0.18) than treated mice (0.78 ± 0.18), a difference of 0.85 ± 0.26 points (p = 0.0037). At day 7, this difference in DAI persisted between treatment groups (0.98 ± 0.30, p = 0.0027). By day 8, disease scores had largely subsided in both groups. However, the transient separation at mid-recovery indicates that the treatment significantly accelerated recovery from DSS-induced colitis. The apparent FITC-dextran translocation in serum decreased significantly after polymer treatment (Figure S7).

To gain insight into the molecular changes associated with these clinical improvements, we performed RNA sequencing on whole-blood clots collected on day 8. Whole blood was chosen because it reflects systemic and immune-driven inflammatory responses. Polymer treatment was associated with reduced expression of transcripts linked to neutrophil activity and matrix degradation (Fig. 7C), consistent with attenuation of inflammatory processes associated with barrier disruption. For example, *Ms4a8a* (log_2_FC = −1.72, FDR = 0.025) is a leukocyte surface signaling molecule that was downregulated. Expression of *Il1r2* (log_2_FC = −2.58, FDR = 0.052), a decoy receptor that sequesters IL-1, was also suppressed, suggesting reduced inflammatory signaling. *Lrg1* (log_2_FC = −2.78, FDR = 0.052), a glycoprotein induced during inflammation that modulates TGF-β signaling and angiogenesis, showed similar decreased gene expression. We also observed lower expression of *Chi3l1* (log_2_FC = −2.95, FDR = 0.059), a chitinase-like protein associated with macrophage activation, fibrosis, and chronic inflammation. Additionally, the gene for *Mmp8* (log_2_FC = −2.52, FDR = 0.059), a neutrophil collagenase that breaks down extracellular matrix and facilitates immune infiltration, was strongly downregulated. Collectively, these results indicate coordinated suppression of transcripts associated with neutrophil-driven inflammation, extracellular matrix remodeling, and systemic inflammatory responses in polymer-treated mice.

We next performed GSEA to determine immune pathways modulated by treatment in DSS-exposed mice (Fig. 7D). This analysis revealed significant downregulation of inflammatory and leukocyte-related processes in whole-blood samples collected at day 8. Pathways associated with neutrophil and myeloid cell activity were significantly suppressed. For example, “leukocyte chemotaxis” (NES = −2.23, FDR = 9.09e-8) and “myeloid leukocyte activation” (NES = −2.12, FDR = 9.09e-8) were significantly negatively enriched in treated mice. Cytokine production pathways were also significantly downregulated. Specifically, “interleukin-6 production” (NES = −2.16, FDR = 1.64e-6) and “regulation of TNF superfamily cytokine production” (NES = −2.08, FDR = 3.32e-6) were reduced. These results indicate attenuation of inflammatory transcriptional programs, particularly those related to leukocyte recruitment and pro-inflammatory cytokine signaling, in polymer-treated mice during DSS recovery.

To interpret mechanisms underlying treatment-induced transcriptional changes, we applied IPA to predict upstream regulators based on the blood expression profile (Fig. 7E). Upstream regulator analysis predicted inhibition of multiple pro-inflammatory regulators. IL1B (Z = −6.86, p = 2.36e-17), TNF (Z = −5.84, p = 3.78e-15), and IFNG (Z = −5.72, p = 2.37e-8) were among the most strongly suppressed, alongside IL6 (Z = −4.02, p = 3.33e-11), IL18 (Z = −3.44, p = 6.86e-7), and IL33 (Z = −2.71, p = 3.15e-14). Regulators of neutrophil and myeloid cell activation were also inhibited, including CSF3 (Z = −3.72, p = 4.55e-11) and CXCL12 (Z = −2.96, p = 4.43e-2). The chemical drivers of acute colitis, dextran sulfate (Z = −8.40, p = 7.16e-19) and lipopolysaccharide (Z = −8.40, p = 7.16e-19), were identified as strongly inhibited upstream regulators. In contrast, two activated regulators, which were IL1RN (Z = 2.58, p = 5.72e-3), an IL-1 receptor antagonist, and SCGB1A1 (Z = 2.00, p = 1.47e-3), a secretoglobin with known anti-inflammatory properties in airway and mucosal immunity, were predicted to be activated. To visualize how several of these regulators interact, we constructed an IPA network centered on lipopolysaccharide (Fig. 7F). The network highlights coordinated inhibition of upstream pattern-recognition receptors, including TLR4 and TLR2, as well as downstream transcriptional drivers such as NFKB1, RELA, and STAT1. Pro-inflammatory cytokines, including IFNG and mediators of NF-κB and STAT signaling, were predicted to be inhibited. Collectively, these findings indicate that, following DSS exposure, polymer treatment was associated with predicted suppression of the LPS-TLR-NF-κB signaling axis and its downstream cytokine programs, alongside activation of counter-regulatory pathways.

**Figure 8:**
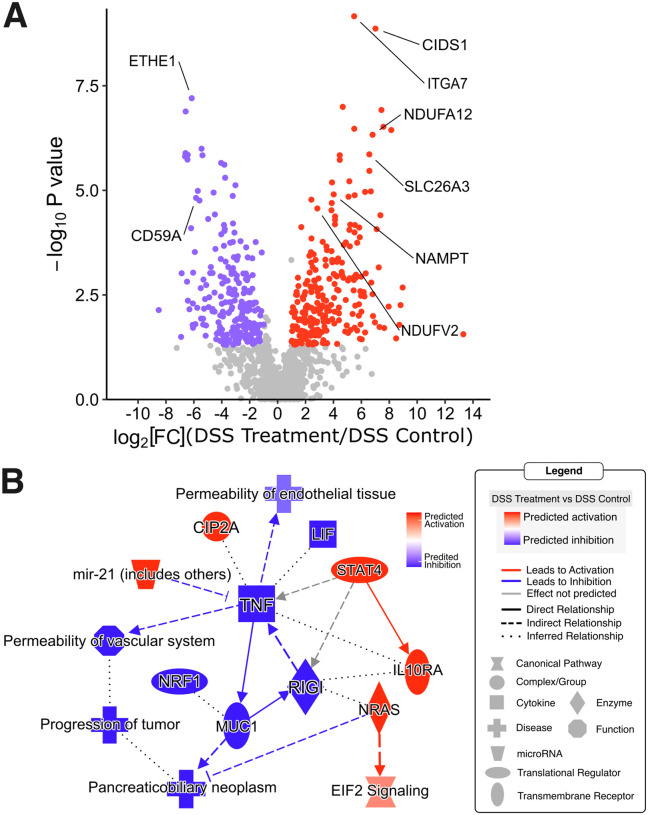
Polymer treatment alters colonic proteomic signatures associated with inflammatory regulation and epithelial homeostasis during DSS-induced colitis. **A**. Volcano plot showing differentially abundant proteins in colonic tissue collected on day 8 from polymer-treated DSS mice relative to DSS controls. Selected proteins associated with epithelial function (red) and inflammatory regulation (purple) are highlighted. **B**. Ingenuity Pathway Analysis (IPA)-derived interaction network based on the colonic proteomic dataset comparing polymer-treated DSS mice and DSS controls. Node color reflects predicted activation state, with red indicating predicted activation and blue indicating predicted inhibition relative to DSS controls. The network highlights predicted suppression of inflammatory and vascular permeability-associated signaling pathways following polymer treatment.

To extend the transcriptional findings, we next profiled the colonic proteome in DSS-treated mice with or without treatment. Several proteins critical for epithelial barrier integrity, mitochondrial, and immune regulation were significantly affected (Fig. 8A). The chloride/bicarbonate exchanger SLC26A3, often depleted in colitis, was upregulated with treatment (log_2_FC = 6.57, FDR = 1.17e-4), while the integrin ITGA7, essential for epithelial repair and adhesion, was similarly induced (log_2_FC = 5.48, FDR = 8.80e-7). In contrast, immune-regulatory molecules including CEACAM1 (log_2_FC = 5.08, FDR = 5.40e-4) and the complement inhibitor CD59A (log_2_FC = −5.84, FDR = 5.62e-4) were differentially expressed. Mitochondrial recovery was evident with induction of Ndufa12 (log_2_FC = 6.80, FDR = 6.07e-5), NDUFV2 (log_2_FC = 2.85, FDR = 8.99e-4), and NAMPT (log_2_FC = 4.02, FDR = 5.22e-4), alongside oxidative stress adaptation via CISD1 (log_2_FC = 7.01, FDR = 8.80e-7). Moreover, the sulfide detoxification enzyme ETHE1 was suppressed (log_2_FC = −6.15, FDR = 2.70e-5), suggesting a metabolic shift that limits mitochondrial overload. Overall, polymer treatment was associated with coordinated changes in epithelial transporters, mitochondrial proteins, and immune-related factors in the colon during DSS recovery, consistent with reduced inflammatory burden and altered epithelial metabolic states.

Proteomic analysis, combined with IPA upstream regulator analysis, highlighted a network centered on TNF signaling (Fig. 8B). Within this network, TNF was strongly indicated to be inhibited (Z = −2.43, p = 5.74e-10), linking directly to reduced endothelial and vascular permeability, as well as downstream suppression of mediators such as STAT4 and IL10RA. LIF, another cytokine positioned within the network, was also suggested to be inhibited (Z = −2.15, p = 1.90e-2), reinforcing the attenuation of pro-inflammatory signaling cascades. Together, this network analysis suggests suppression of classical cytokine-driven inflammatory programs in the colon during DSS recovery in polymer-treated mice.

## DISCUSSION

Disruption of the intestinal mucus barrier and the resulting loss of epithelial-microbial segregation are increasingly recognized as contributors to gastrointestinal and systemic disease, as they allow bacteria or pathogen-associated molecular patterns (PAMPs) to access the epithelium, driving immune activation and tissue injury^29–31^. Recent experimental evidence suggests that excessive mucus secretion may protect against colitis^32^. However, current therapies for alleviating intestinal inflammation do not directly address epithelial luminal interactions. Mucosal healing, when observed, typically occurs as a downstream consequence rather than an active targeted repair process^33,34^. In this study, we examined the in vivo effects of an orally administered polymer formulation in *Agr2*^−/−^ mice generated by Park et al.^24^, which exhibit impaired mucus production, and in the Dextran Sulfate Sodium (DSS)-induced colitis model of acute epithelial injury established by Okayasu et al^35^. Our goal was to evaluate whether an intervention aimed at improving epithelial-microbial segregation could attenuate inflammatory responses across these complementary models.

To enable in vivo evaluation of this polymer, we first assessed whether repeated oral delivery could be achieved without adverse effects. Short-term oral administration of the polymer was well tolerated in wild-type mice, with no clinical abnormalities, changes in water intake, body weight, or microbiota composition observed over the exposure period. These findings indicate that repeated luminal exposure to the polymer is feasible in vivo. However, the present study was not designed to evaluate long-term safety, pharmacokinetics, or chronic dosing, which will be important considerations for future translational studies. Because the polymer was administered orally, the material necessarily transits through the stomach and small intestine prior to reaching the colon. The present study was designed to determine site-specific interactions within the small intestine and colon, preventing any conclusions regarding polymer activity in other regions. MALDI mass spectrometry imaging localization experiments using unlabeled polymer suggest that the polymer predominantly localizes within the lumen. Importantly, localization was assessed using a label-free approach, which minimizes the concerns that fluorescein conjugation alters polymer function, retention, or physicochemical interactions in vivo. In conjunction with fecal recovery experiments, the data indicate that the material is not permanently retained in the gastrointestinal tract and is cleared upon cessation of treatment, supporting transient luminal exposure. The longer persistence of the polymer signal in the colon may arise from slower colonic transit and local physicochemical conditions. In particular, boronic acid-diol interactions are pH-sensitive, raising the possibility that regional pH differences modulate polymer retention. This mechanism will require direct testing.

Across both models, the biological conclusions were based on the consistency of the observed proteomic signatures and their agreement with multiple orthogonal measurements, including histological assessments, permeability measurements, blood transcriptomics, and microbiome profiling. Improvements of epithelial-microbial segregation were consistently accompanied by attenuation of innate immune signaling, including NF-κB, interferon, and TNF-associated pathways, with concordant findings across the gut microbiome. This convergence supports the hypothesis that immune modulation arises predominantly as a secondary consequence of improved epithelial-microbial segregation rather than direct immunosuppression. Nevertheless, while our data favor this indirect model, we cannot fully exclude the possibility that the polymer exerts minor direct effects on epithelial or immune signaling pathways. Dissecting contributions will require future studies using barrier-independent immune challenges.

In both mouse models of compromised epithelial-microbial segregation, treatment was associated with reduced hallmark symptoms of colitis, including weight loss, diarrhea, and fecal blood. In the *Agr2*^−/−^ model, treated mice gained body weight to levels comparable to those of age-matched WT controls and exhibited approximately a 50% reduction in colitis score by day 4, indicating rapid and sustained improvement in disease severity. Histological analysis further revealed reduced hyperplasia, diminished infiltrating leukocytes, and partial restoration of crypt organization, consistent with improved epithelial homeostasis. The intestinal mucus layer plays a central role in host defense by maintaining spatial separation between luminal microbes and the epithelium, thereby limiting epithelial exposure to microbial products and inflammatory triggers^1,36–38^. This function is particularly critical in the colon, where dense microbial communities closely coexist with the host^39^. When the mucus layer is compromised, as in Agr2−/− mice, bacteria gain access to the epithelial surface, triggering a marked inflammatory response. Treatment was associated with restored epithelial-microbial segregation, preventing pathogenic bacteria from directly interacting with the intestinal epithelium and thereby avoiding the activation of pro-inflammatory cascades. Consistent findings were observed in DSS-injured mice, in which treatment accelerated weight gain and DAI recovery. The DSS model (2.5% for 4 d) induced moderate colitis, which captures mucus depletion and epithelial barrier disruption without severe epithelial destruction^40^. Faster resolution of disease symptoms suggests improvements in the epithelial-microbial separation, accompanied by attenuation of downstream inflammation during recovery^41^.

The analyses of transcriptomic and proteomic data in *Agr2*^−/−^ and DSS-induced colitis, including Gene Set Enrichment Analysis, suggested suppression of innate immune response pathways following treatment. The IPA analysis identified LPS as a major upstream driver of these inflammatory cascades in untreated mice. LPS, an abundant component of Gram-negative bacterial membranes, is also released into the lumen during bacterial turnover, where it acts as a potent pro-inflammatory stimulus. In mucus barrier deficiency models, where epithelial-microbial separation is compromised, luminal LPS is more likely to access the epithelial surface, triggering an inflammatory response. Consistent with this interpretation, loss of epithelial-microbial segregation increases the probability of direct epithelial exposure to bacterial products such as LPS.

In many bacterial species, lipopolysaccharide (LPS) and other surface-associated molecules, including flagellin, contain glycan structures enriched in sialic acid residues^42^. These glycans may contribute to molecular mimicry strategies that promote microbial persistence and biofilm formation^42^. Sialic acids contain cis-diols^43^, which are known to form reversible covalent interactions with boronate groups^44–47^. Phenylboronic acid derivatives have been reported to exhibit affinity for specific carbohydrates, including N-acetylneuraminic acid (sialic acid), at physiological pH^48^. Because the polymer used in this study incorporates phenylboronic acid functionality, it may form transient interactions with cis-diol-containing biomolecules in the lumen, including bacterial glycoconjugates, host glycocalyx components, and soluble glycoproteins. Although direct in vivo binding or sequestration of LPS was not directly demonstrated in this work, the in vitro LPS retention assay (Fig. 1H) provided an indication that boronic acid incorporation enhances LPS retention relative to the polymer backbone alone. Notably, the PDMA backbone lacking APBA also reduced the LPS translocation rate, suggesting that nonspecific physicochemical mechanisms contribute to LPS translocation reduction. The high molecular weight of the polymer (Fig. 1B) may promote chain entanglement, forming a polymer mesh that restricts LPS diffusion. Hydrophilic polymers form strongly hydrated interfacial layers that create steric repulsion to protein and bacterial adhesion^49–51^. Acrylamide-based polymers such as poly(*N,N*-dimethylacrylamide) (PDMA) exhibit high water affinity due to the presence of amide functional groups that can form hydrogen bonds, enabling the formation of highly hydrated polymer domains without loss of structural integrity^52^. These hydration effects, combined with the high molecular weight of the polymer and the transient viscosity (Fig. 1D), might contribute to reducing epithelial exposure to bacterial toxins by slowing the diffusion of macromolecules such as LPS. These findings support a model in which reversible boronate-diol interactions and the hydrophilic polymer characteristics might both contribute to reducing epithelial exposure to pro-inflammatory microbial products. While the polymer can potentially bind to native mucins, the improved reduction of epithelial exposure to luminal microbial products observed in *Agr2*^−/−^ mice treated with polymer suggests that secreted mucins are not strictly required for polymer adhesion. Interactions with alternative cis-diol-containing structures (e.g., the epithelial glycocalyx and membrane glycoproteins) may also contribute to polymer adhesion.

Consistent with reduced epithelial exposure to bacterial products, a granular analysis of protein expression enabled us to elucidate the molecular mechanisms by which the innate immune system is modulated by treatment across both models. This revealed a coherent pattern centered on the canonical NF-κB and interferon pathways. In *Agr2*^−/−^ treated mice, we observed significant downregulation of colonic Lipopolysaccharide-binding protein (LBP) and splenic CD14 proteins. Mechanistically, LBP functions as a soluble pattern recognition protein that binds LPS and transfers it to the co-receptor CD14, which presents the ligand to Toll-like receptor 4 (Tlr4)^53^. TLR4 is also activated by calprotectin, which recognizes damage patterns to amplify an innate immune response in the colon^54^. For this reason, calprotectin is also a robust marker for colitis^55,56^. This LPS-driven interaction normally recruits and activates the IκB kinase (IKK) complex, including the IKKβ and IKKe subunits, leading to phosphorylation of IkB and subsequent release of NF-κB^57^. The release of NF-κB transcription factors drives pro-inflammatory gene expression of cytokines such as TNF-α^58^, which IPA predicted to be inhibited in the colon of *Agr2*^−/−^ treated mice. The reduction of LBP, CD14, IKKβ, IKKe, and calprotectin observed in *Agr2*^−/−^ treated mice, therefore, interrupts this signaling cascade and is consistent with the suppression of the NF-κB p50 subunit (NFKB1) seen in the colon. Additional activators of NF-κB were also reduced in the spleen of *Agr2*^−/−^ treated mice, such as NOD1, a sensor of bacterial peptidoglycan^59,60^, and IL1RAP, an accessory protein for IL-1 receptor signaling^61,62^.

Beyond NF-κB, Tlr4 engagement by LPS initiates a TRIF-dependent signaling branch that induces type I interferons^63^. Consistent with this branch, our colon proteomic data from *Agr2*^−/−^ treated mice showed reduced abundance of the interferon-inducible protein GBP2^64^. At the same time, IPA suggested inhibition of upstream regulators IFN-γ, IFN-α, and IFN-β. This dual attenuation of NF-κB and interferon signaling is significant because these convergent pathways sustain chronic mucosal inflammation, drive epithelial injury, and delay barrier recovery, as reviewed previously^65,66^. In the absence of mucus, *Agr2*^−/−^ mice exhibit chronic overstimulation of pattern-recognition pathways, resulting in NF-κB and interferon-mediated inflammation that perpetuates epithelial damage and fosters a pro-inflammatory microenvironment.

In the DSS model, similar results further suggest improved epithelial-microbial segregation, followed by recalibration of innate immunity. In the colon of DSS-injured mice, NAMPT was increased and can influence NF-κB signaling directly as a putative damage-associated molecular pattern ligand and indirectly via NAD-dependent pathways^67,68^. In DSS-injured mice treated with the polymer, several blood transcripts associated with NF-κB activation and inflammatory leukocyte recruitment (*Il1r2, Chi3l1, Lrg1*, and *Mmp8*) were downregulated relative to DSS controls. These genes contribute to leukocyte recruitment, tissue remodeling, and chronic mucosal inflammation^69–72^. The colon proteome similarly indicated attenuation of inflammatory signaling pathways, including predicted inhibition of TNF-α as an upstream regulator, consistent with reduced NF-κB-associated inflammatory activity during recovery. Similar to *Agr2*^−/−^, suppression of interferon signaling through inhibited upstream regulators IFNG and STAT1 was indicated by IPA of the blood transcriptome in DSS-injured mice recovering with treatment.

Furthermore, NR3C1 (glucocorticoid receptor, GR) and STAT5A were predicted to be activated by IPA. Upon binding to ligands (like cortisol), GR acts as a potent anti-inflammatory transcription factor. It also directly inhibits NF-κB and AP-1 (transrepression), consequently blocking the transcription of cytokines such as IL-1β, TNF-α, and IL-6. STAT5A activation is essential for intestinal stem cell proliferation and regeneration after injury^73^. This suggests that the treatment shifted from an acute inflammatory state to a regenerative or homeostatic program focused on tissue recovery and the resolution of inflammation. Taken together, this comprehensive blockade of the NF-κB pathway likely accounts for the observed attenuation of TNF-alpha cytokine production in both models and for dampened leukocyte recruitment, underscoring the central role of NF-κB signaling in the pathogenesis and potential treatment of colitis.

One downstream consequence of leukocyte recruitment and immune activation in colitis is the disruption of epithelial hypoxia, excessive production of reactive oxygen species (ROS), and impaired mitochondrial function. Key players in the NF-κB pathway, such as NF-κB and calprotectin, promote ROS release, which is amplified by neutrophil recruitment^74,75^. These markers were significantly reduced in *Agr2*^−/−^ treated mice, indicating attenuation of inflammatory oxidative stress. In the DSS model, proteomic evidence of improved mitochondrial and redox metabolism, such as increased NDUFA12^76^, NDUFV2^76^, CISD1^77,78^, ETHE1^79,80^, and NAMPT^81,82^, supports the reestablishment of mitochondrial oxygen consumption and redox homeostasis. These highlight critical improvements from a pathological microenvironment, as excess ROS and disrupted oxygen balance impede mucosal repair.

Colitis-driven mitochondrial dysfunction elevates tissue oxygenation, disrupting the hypoxic niche that typically supports obligate anaerobes^83^. The disruption of anaerobiosis and reduced microbial diversity are considered indicators of dysbiosis^84,85^. Increased representation of aerobic respiration pathways in the microbiota of DSS colitis mice suggests regenerative hyperplasia and enhanced oxygen diffusion into the lumen^86^. In *Agr2*^−/−^ treated mice, anaerobiosis, indirectly measured by the ratio of obligate to facultative anaerobes, and microbial diversity were significantly improved. Increased abundance of short-chain fatty acid (SCFA)-producing taxa, including *Roseburia hominis, R. intestinalis*^87^, *R. faecis*^87^, *Anaerotruncus colihominis*^88^, *Oscillibacter valericigenes*^89^, and *Alistipes communis*^90^, was observed only in *Agr2*^−/−^ treated mice. SCFAs benefit the host by fueling epithelial cells, reinforcing barrier integrity, tempering inflammation, and regulating lipid metabolism^91^. Conversely, pathogenic facultative anaerobes such as *Klebsiella variicola* and *Klebsiella pneumoniae*, both associated with colitis exacerbation^92^, were reduced. This microbial modulation may reflect improved luminal ecosystem conditions associated with improved epithelial-microbial separation, while potential direct interactions between the polymer and bacterial surface glycans via boronate chemistry cannot be excluded.

This work shows that oral administration of a polymeric mucus surrogate is associated with improved clinical and histological outcomes and broad attenuation of inflammatory programs in both a genetic model of mucus deficiency and a DSS injury model. Across models, treatment was consistently associated with reduced epithelial-associated bacterial signals, shifts toward improved anaerobic microbial community traits, and suppression of innate immune pathways. These patterns align with the principle of a healthy colonic mucus system, which is based on reduced exposure of luminal contents to the epithelium when epithelial-microbial separation is effective. Collectively, these findings support the feasibility of barrier-directed, non-immunosuppressive strategies to modulate intestinal inflammatory responses. They also underscore the need for future studies to quantitatively assess polymer interactions within the intestinal lumen, spatial retention dynamics, and long-term exposure.

## METHODS

### Polymer design and characterization

Polymers were synthesized via photoiniferter polymerization. To a 40 mL scintillation vial, *N,N*-dimethylacrylamide (DMA; 11.6 mL, 113 mmol, 13600 equiv), 3-acrylamidophenylboronic acid (APBA; 1.14 g, 5.96 mmol, 719 equiv.), and 2-(Dodecylthiocarbonothioylthio)-2- methylpropanoic acid (DDMAT; 33.6 mg, 0.00828 mmol, 1 equiv.) were dissolved in dimethyl sulfoxide (DMSO; [4M]). DMF (3.0 mL) or trioxane (3.0 g) was used as an internal standard to track monomer consumption. The mixture was sparged with Ar for 20 min, and the solution was subjected to UV light (365 nm, 3.5 mW cm^2^) at room temperature for 10–24 h until full monomer conversion was observed by ^1^H nuclear magnetic resonance (NMR). *N,N-*Dimethylacrylamide was passed through a column of basic alumina (Al_2_O_3_, Sigma, standard grade/Brockmann I/activated) to remove inhibitors prior to polymerization. The C12 iniferter, 2-(dodecylthiocarbonothioylthio)-2-methylpropanoic acid (DDMAT), was synthesized according to previous literature protocols^93,94^. The UV light source used was a MelodySusie nail lamp.

Fluorescently labeled polymers were synthesized using methods similar to those outlined above. Fluorescein O-methacrylate (FMA; 20 equiv.) was added with the monomer, iniferter, solvent, and internal standard to the reaction vial. The mixture was sparged with Ar for 20 min, and the solution was subjected to UV light (365 nm, 3.5 mW cm^2^) at room temperature for 10–24h until full monomer conversion.

For polymer purification, crude polymers were dissolved in water and purified by dialysis (RC, 15 kD pore size) in 4 L of solvent, with the solvent changed 10 times (40 L). The purified polymer was dried via lyophilization (< 0.300 bar, −78°C) for 72 h. Purity was determined through ^1^H NMR, where the disappearance of DMSO and monomeric vinyl traces indicated purity.

### Size Exclusion Chromatography (SEC)

SEC was performed in *N,N*-dimethylacetamide with 50 mM LiCl at 50°C and a flow rate of 1.0 mL/min (Agilent isocratic pump, degasser, and autosampler; columns: Viscogel I-series 5 μm guard + two ViscoGel I-series G3078 mixed bed columns, molecular weight range 0 − 20 and 0 − 10,000 kg/mol). Detection consisted of Wyatt Optilab T-rEX refractive index detector operating at 658 nm and a Wyatt miniDAWN TREOS light scattering detector operating at 690 nm. Molecular weight distributions were calculated using the Wyatt ASTRA software. For fluorescently tagged polymers where light scattering traces were undeterminable, molecular weight distributions were calculated using a PMMA conventional calibration.

#### ^1^ H Nuclear Magnetic Resonance (NMR)

Spectra were recorded on an INOVA 400 MHz spectrometer and processed in MestreNova software. The percentage of APBA was calculated by integration of phenyl protons against methylene (-CH2-) protons. Chemical shifts (δ) are given in parts per million (ppm).

### Thermal gravimetric analysis (TGA)

TGA experiments were run on a TA 5500 equipped with an autosampler using a 100 μL platinum pan. The ramp rate heated was at 5°C/min from 25–500°C under nitrogen flow (10 mL/min).

### Dynamic light scattering (DLS)

Dry polymers were dissolved overnight in DI water or PBS buffer solutions (pH = 1.6 or 6.7) in 5 mg/mL concentrations. The polymer solutions were passed through a 0.45 μm nylon filter to remove debris, prepared in PMMA cuvettes, and analyzed with a Malvern Zetasizer Nano ZS at room temperature. DLS measurements were recorded in triplicate using Zetasizer software, and the particle size averages were plotted against normalized intensity in Origin.

### Shear rheology

All shear rheology was recorded on a DHR-2 rheometer and plotted using Origin software. Solutions were mediated at 25°C under a metal cover slip, and the circumference of the cover slip was wet with water to seal in moisture. Each experiment type was run in triplicate.

Shear Rate. Shear rate dependence experiments utilized a 20 mm parallel plate geometry, with parameters set to 20 Pa stress and 0.1—100 Hz to reflect relevant gastrointestinal cell clearance rates. 0.3–0.4 mL of a polymer solution (HPLC water, 5 wt%) was aliquoted per experiment.

Angular Frequency. Frequency dependence experiments utilized a 40 mm cone geometry, which reduced sample slippage observed in oscillatory stress as compared to the standard 20 mm parallel plate. Parameters were set to 20 Pa stress and 50—0.1 rad/s at 25°C. 0.7–0.9 mL of a polymer solution (HPLC water, 5 wt%) was aliquoted per experiment, and the experiment was run in triplicate. Stress-dependence tests utilized a 20 mm parallel plate geometry, with parameters set to 1 Pa stress for most of the experiment’s duration. At select time points (t = ~ 120 and 300 s), the stress was briefly increased to 300 Pa before relaxing back to 1 Pa.

### Epithelial-bacterial coculture assay

Cells from a human telomerase-immortalized corneal epithelial (hTCEpi) cell line were thawed and cultured as previously described^95^. Glass-bottom (#00 coverslip) imaging dishes were seeded at 0.5 million cells per mL, with cells stained with CellTracker^™^ Deep Red (Invitrogen^™^ C34565). The dish was cultured for 2 d to reach confluence before the experiments. The *E.coli* (Castellani and Chalmers, 25922^™^) were separately cultured and stained with BactoView^™^ Live Red (Biotium^™^, 40101-T). The fully confluent dishes were incubated for 1 h with a solution containing 1.5% wt polymer labeled with Fluorescein acrylamide, and then the *E. coli* suspension was added to the dishes. The image was captured by confocal microscopy on a Nikon AX-R confocal microscope.

### Polymer spatial localization and retention time

Polymer localization and gastrointestinal retention were evaluated in healthy C57BL/6J mice (8 weeks of age). Animals were randomly assigned to treatment or control groups (n = 3 per group per time point). The treatment group received polymer dissolved in drinking water (6.60 mg/mL), while controls received drinking water without polymer. Mice were treated for 48 h, after which polymer administration was discontinued. Tissue collection was performed immediately at the end of treatment (48 h from experiment initiation), 48 h after treatment cessation (96 h from experiment initiation), and 5 d after treatment cessation (7 d from experiment initiation). Duodenal and colonic tissues were collected, flash-frozen in liquid nitrogen, and stored at −80°C until cryosectioning and label-free MALDI mass spectrometry imaging (MALDI-MSI) analysis.

Intestinal tissue sections were analyzed using matrix-assisted laser desorption/ionization mass spectrometry (MALDI-MS) and MALDI mass spectrometry imaging (MALDI-MSI) to evaluate polymer-associated spectral features and their spatial distribution within tissue. The analytical workflow consisted of two sequential steps: (i) characterization of the polymer standard to determine its ionization behavior and identify candidate polymer-associated m/z features, followed by (ii) spatial analysis of selected ions in intestinal tissue sections. For polymer characterization, the polymer standard was prepared at 1 mg/mL in ultrapure water and mixed separately with different MALDI matrices to assess ionization performance under both ionization polarities. α-Cyano-4-hydroxycinnamic acid (CHCA) was used for positive ion mode, while N-(1-naphthyl)ethylenediamine dihydrochloride (NEDC) was used for negative ion mode. Aliquots (3 μL) of each polymer–matrix mixture were spotted onto a MALDI MTP384 target plate and dried under vacuum desiccation to promote uniform crystallization.

Initial MALDI-MS screening was performed to distinguish polymer-derived signals from matrix background ions. Spectra were acquired in MS mode over an m/z range of 20–1300 using signal summation from multiple laser positions across the spot (50 μm laser focus, 400 laser shots per position, 1000 Hz repetition rate, ~ 75% laser power). Acquired spectra were processed using Bruker DataAnalysis software, and candidate polymer-associated ions were selected based on reproducible detection in polymer-containing spots while being absent from matrix-only controls.

For tissue imaging experiments, mouse intestinal and colonic tissues were cryosectioned (10 μm thickness) and mounted onto indium tin oxide (ITO)-coated conductive glass slides. A polymer reference spot was deposited adjacent to the tissue section on the same slide to enable direct spectral comparison under identical acquisition conditions. Matrix was applied uniformly using an automated HTX sprayer at 60 μL/min with a tray temperature of 50°C using NEDC (7 mg/mL in 70% methanol). Although both ionization modes were initially evaluated, the polymer showed superior signal intensity and reproducibility in negative ion mode; therefore, subsequent imaging analyses were performed using NEDC under negative ion conditions.

MALDI-MSI data were acquired using a Bruker timsTOF fleX instrument operated in MALDI mode with TIMS disabled, in negative ion MS acquisition mode over an m/z range of 20–1300. Instrument transfer settings included a MALDI plate offset of 30 V, deflection delta of − 60 V, funnel RF settings of 200 Vpp, collision RF of 700 Vpp, transfer time of 80 μs, and collision energy of 7 eV. Spectra were acquired using raster imaging at 20 μm spatial resolution. Imaging data were processed using SCiLS Lab software. Ion distribution maps were generated for selected candidate m/z values and compared across tissue regions, polymer reference spots, and matrix control regions. Candidate polymer-related ions were defined as features consistently detected in both polymer standards and tissue sections, while absent from matrix-only controls and untreated tissue samples. Spatial localization patterns were evaluated to assess potential polymer presence or tissue interaction.

To complement tissue localization studies, polymer clearance was independently monitored in fecal samples from healthy 8-week-old C57BL/6J mice using fluorescein-labeled polymer. Mice were treated with different doses of polymer dissolved in autoclaved water. Fecal pellets were collected at multiple time points during and after treatment to assess temporal clearance patterns. Fecal samples were collected and homogenized in 200 μL PBS. After homogenization, samples were centrifuged at 5,000 RPM for 5 minutes. The supernatant was then transferred to a black Costar 96-well plate and analyzed using the Cytation^™^3 instrument (BioTek^®^) (excitation 487 nm/emission 528 nm). Fluorescence readings were normalized by dividing the raw fluorescence units (FU) by their respective control.

### Cryogenic scanning electron microscopy

Hydrated polymer samples prepared in PBS were cryogenically preserved for ultrastructural imaging. Samples were frozen in liquid nitrogen, fractured under cryogenic conditions to expose internal microarchitecture, and transferred to a cryogenic preparation chamber for controlled sublimation and conductive sputter coating. The cryo-preservation workflow was selected to minimize dehydration-induced collapse and preserve hydrated network organization. Imaging was performed using a Hitachi SU5000 scanning electron microscope equipped for cryogenic analysis and operated at 5.0 kV using secondary electron detection. Images were acquired at 5,000× magnification with a working distance of 8.6–8.9 mm.

### Lipopolysaccharide (LPS) diffusion assay

The polymer’s capacity to retain bacterial lipopolysaccharide (LPS) was evaluated in vitro using a transwell plate system. The assay was conducted at pH 7.8 and 6.5 to assess pH-dependent contributions of boronic acid-diol interactions. Measurements of FITC-LPS translocation across a dialysis membrane were collected at 24-hour intervals for up to 96 hours. To evaluate the role of boronic acid functionality, two polymer formulations were tested: (i) an original polymer preparation composed of hydrophilic *N,N*-dimethylacrylamide (DMA) repeat units incorporating 3-acrylamidophenylboronic acid (APBA), and (ii) a control polymer consisting of the PDMA backbone without APBA.

Polymers (6.60 mg/mL) were prepared in phosphate-buffered saline (PBS) adjusted to pH 7.8 or 6.5 and loaded into 0.4 μm transwell inserts (Corning, Catalog #3460). The exterior of each insert was fitted with a dialysis membrane (Fisher, Catalog #08–667E) to retain the polymer while allowing diffusion of unbound FITC-LPS. Five micrograms of FITC-LPS (Sigma-Aldrich, Catalog #F3665) were added to the apical compartment. At 24, 48, 72, and 96 hours, samples were collected from the basal compartment, and fluorescence was measured at 528 nm (excitation 487 nm). LPS concentrations were determined using a standard calibration curve generated from known FITC-LPS concentrations. Each condition was tested in five independent replicates.

Differences among conditions were tested using one-way ANOVA, followed by Tukey’s post hoc test to adjust for multiple comparisons and determine specific pairwise group differences. Effect sizes were calculated using Hedges’ g to estimate the magnitude of differences between groups.

### In-vivo tolerability

Short-term in-vivo tolerability was assessed in healthy C57BL/6J mice (12 ± 3 weeks old). Animals were randomized into groups (n = 3 per treatment) that received the polymer dissolved in autoclaved water at concentrations of 0.40, 0.80, 1.60, 2.50, and 6.60 mg/mL, along with an untreated control group that received water only for 10 d. Based on measured water consumption and body weight, the estimated polymer intake across dose groups was approximately 100 ± 26, 199 ± 53, 398 ± 106, 602 ± 160, and 1600 ± 426 mg/kg/day, respectively. The administered concentrations were chosen to span a range predicted to provide increasing intestinal surface coverage based on published estimates of murine intestinal surface area^96^. To estimate daily intake, water bottles were weighed daily, and consumption was calculated as the difference in weight between the initial and final weights.

#### Mucus-deficient Agr2^−/−^ mice model

The knockout of anterior gradient 2 (*Agr2*) by exon 2–3 deletion results in the misfolding and secretory inhibition of MUC2, the dominant intestinal mucin^24^. Wild-type (7 weeks) and *Agr2*^−/−^ (8 ± 2 weeks) littermates were randomized into control and treated groups (n = 7–9 per treatment) and received 6.60 mg/mL polymer in drinking water for 17 d. Based on measured water consumption and body weight, the estimated polymer intake was approximately 1750 ± 624 mg/kg/day in *Agr2*-treated mice and 1680 ± 590 mg/kg/day in treated wild-type mice. The dose was selected empirically as a well-tolerated concentration that ensured continuous luminal exposure throughout the gastrointestinal tract while remaining below levels associated with reduced water intake or clinical intolerance.

The generation of *Agr2* knockout mice has been described previously^24^. Briefly, the heterozygous knockouts purchased from Jackson Laboratory (B6.129S4(FVB)-Agr2 < tm1.2Erle>/J, strain: Strain #:025630) were interbred by the UF Rodent Models Breeding Core to produce experimental homozygous KO and wild-type littermate control mice. Genotyping for *Agr2* KO mice was performed by PCR using tail DNA and a set of 20629 (5′- GGT TTG GGC CTG AAA CTC TG -3′), 20630 (5′- ACC ATC AAG GGT CTG TTG CT -3′), and 20631 (5′- GGC CAT GGG TAC CTT TAG TG -3′) primers. The expected PCR product for the mutant is a single 400 bp band. For a heterozygous individual, there are two bands, one of 252 bp and another of 400 bp. Wild-type mice produce a single band of 252 bp.

### Dextran Sulfate Sodium (DSS)-induced colitis model

To create the acute DSS model, we followed the basic protocol described by Chassaing et al.^27^. Briefly, wild-type mice (8 weeks old) were randomized into control (n = 6: 3 females and 3 males) and treatment (n = 6: 3 females and 3 males) groups. Both groups received 2.5% (w/v) dextran sulfate sodium (DSS) in drinking water for 4 d to induce colitis. Weight and colitis symptoms were monitored daily. On days 4–8, DSS administration was discontinued; the control group received autoclaved water, while the treatment group received polymer (6.60 mg/mL) dissolved in water. The polymer intake was approximately 1675 ± 464 mg/kg/day.

### Body Composition

Body weight was recorded every two days, and stool consistency and bleeding were recorded every four days. Upon completion of the study, control and treated *Agr2*^−/−^ carcasses were subjected to a Dual-energy X-ray absorptiometry (DEXA) scan performed by the Malcom Randall Department of Veterans Affairs Medical Center. A linear mixed-effects model (LME) was employed for analyzing longitudinal data, such as repeated measures of body weight from the same animals. LMEs account for fixed effects (treatment and time) and random effects (individual differences among mice), providing more precise and reliable results compared to simpler models, such as ANOVA, which do not effectively handle within-subject correlations. The post hoc Estimated Marginal Means (EMM) analysis, implemented using the emmeans package v1.10.3^97^, was applied to conduct detailed pairwise comparisons between different treatment groups and time points, further clarifying the specific differences detected by the LME.

### Clinical Disease Activity Scoring

For the *Agr2*^−/−^ model, colitis scores were recorded every four days following the adapted criteria of Rodrigues et al.^98^ to enable longitudinal assessment of disease progression. In this scoring system, a score of 0 represented < 4% weight loss, normal stool, and no fecal blood; 1 = 5–10% weight loss, soft stool, and no fecal blood; 2 = 11–20% weight loss, loose stool, and trace fecal blood; 3 = 21–30% weight loss, diarrhea, and visible blood; and 4 = > 30% weight loss, empty and wet colon, and gross bleeding. For the DSS model, body weight, stool consistency, and fecal bleeding were recorded daily and incorporated into the Disease Activity Index (DAI) as described by Cooper et al.^40^. The DAI integrates these parameters into a composite score reflecting colitis severity. The scoring criteria were as follows: 0 = 0–1% weight loss, normal stool, no fecal blood; 1 = 1–5% weight loss; 2 = 5–10% weight loss, loose stool, trace fecal blood; 3 = 10–15% weight loss; and 4 = 15–20% weight loss, diarrhea, and gross bleeding. Longitudinal trends in DAI and colitis scores were analyzed using LME models with treatment × time as interaction terms, followed by post hoc pairwise contrasts (*emmeans*). Statistical significance was set at FDR < 0.05.

### Histological Assessment

Colonic tissues were dissected from mice following euthanasia. The luminal content of the colon was washed with phosphate-buffered saline (PBS), and the sample was cut longitudinally. Starting from the distal end, with the luminal side facing upward, the colon was rolled into a Swiss roll. The tissue was preserved in 10% Neutral Buffered Formalin, paraffin-embedded, sectioned, and stained with hematoxylin and eosin by the Molecular Pathology Core at the University of Florida. Histological scoring of inflammation was independently and blindly performed by two pathologists according to the criteria defined in Supplementary Table S11.

### FITC-Dextran assay

The standard FITC-dextran assay (4 kDa) was used to assess intestinal permeability, as described previously^99^. ^103101979899^After fasting for 4 hours, FITC-dextran was orally gavaged, and blood was collected after 4 hours, followed by euthanasia. Fluorescence was determined at 530 nm with excitation at 485 nm from plasma samples (1:10 in 1x PBS) in duplicate. Concentrations were determined using a standard calibration curve generated from known FITC-dextran concentrations.

### RNA In Situ Hybridization (ISH) for bacteria detection

Bacterial 16S ribosomal RNA (rRNA) was detected using the RNAscope^™^ 2.5 HD Reagent Kit-RED (Advanced Cell Diagnostics, Inc., Newark, CA, Cat# 322430) according to the manufacturer’s protocol. Briefly, FFPE sections were deparaffinized in xylene and rehydrated in a graded ethanol series. Slides underwent target retrieval and protease digestion for 30 min at 40°C. Sections were then hybridized with the RNAscope^™^ Probe - EB-16S-rRNA-C2 (ACD, Cat# 464461-C2), which targets a degenerate sequence (16SrRNA) in conserved regions of Eubacteria 16SrRNA. Subsequent signal amplification was performed using the RNAscope HD 2.5 amplification reagents (AMP 1–6). The signal was detected using the Chromogenic HD RED substrate (Fast Red). Slides were counterstained with Gill’s hematoxylin, dehydrated, cleared, and cover slipped using VectaMount mounting medium.

Control probes were run in parallel: RNAscope Probe - PPIB (a medium-expression positive control) and RNAscope Probe - Mm-Clec4f-C2 (C-type lectin domain family 4, member F gene, which is exclusively expressed by Kupffer cells in the liver, serving as a negative control) to ensure signal specificity and tissue RNA integrity (Supplementary Figure S8).

All slides were digitally scanned at 20x in brightfield mode using an Olympus VS200 whole-slide scanner (Olympus Corporation, Tokyo, Japan) located at the University of Florida Molecular Pathology Core. Digital images were viewed and analyzed using OlyVIA software (Olympus).

### Microbiome

Collected fecal samples were immediately preserved at −80°C, and genomic DNA was extracted using the QIAmp Fast DNA Stool Mini Kit (QIAGEN, Germany; Cat No.#51604) according to the manufacturer’s protocol. DNA was quantified using the dsDNA High Sensitivity Assay (Thermo Fisher Scientific, Massachusetts, USA) on the Qubit 4.0. The 16S rRNA gene was amplified using LongAmp Hot Start Taq 2X Master Mix (New England Biolabs, Inc.) with two universal 24-mer-barcoded-primers: 27F (AGAGTTTGATCMTGGCTCAG-3’) and 1492R (CGGTTACCTTGTTACGACTT-3’). The fusion barcode primer was synthesized using the same sequences as the Native Barcoding Kit 96 V14 (SQK-NBD114.96). We utilized a common 24-base barcode for each primer pair. Library preparation adhered to the Ligation sequencing DNA V14 (SQK-LSK114, Oxford Nanopore Technologies) protocol, and the final library was loaded onto an R10.4.1 (FLO-MIN106) flow cell and sequenced using a GridION device for 72 hours. The raw nanopore signal was basecalled using Dorado v5.0.0 on the super high accuracy model, sup. The contingency table, assembled at the bacterial species level, was obtained using the RESCUE pipeline^100^.

The dataset was rarefied to the minimum library size to perform all microbiome analyses as previously recommended^101^. The study design for acquiring the microbiome data consisted of three separate sequencing runs. Thus, the data required examination and correction for any batch effects. A non-parametric batch removal model titled “Conditional Quantile Regression” (ConQuR), available through the ConQuR package in R, was utilized for this purpose^102^. Covariates were then identified as the days, sex, dose, and genotype of mice. These covariates were considered relevant due to their continuous and uncontrolled nature, as well as their ability to define the condition type for each sample. The age of the mice was not included as a covariate, as this variable was intentionally controlled to be within a specific range, thereby negating its potential effect on batch variation. Pairwise PERMANOVA was used to assess the results from the ConQuR analysis of the data, with a p-value of 0.05 serving as the threshold for significance. Due to the absence of significant batch effects in the data, both distance metrics, Bray-Curtis and Aitchison, were used to explore parameter variation, yielding consistent results (Figure S9). This secondary analysis was conducted on control samples only within wild-type mice, followed by PERMANOVA statistical analysis to examine the differences.

Alpha diversity was calculated using the vegan package (v2.6–6.1) with the Shannon diversity index^103^. The Shapiro-Wilk test was used to determine the normality of the Shannon diversity index. Beta diversity was examined using PCoA. Variables influencing beta diversity were tested using Permutational Multivariate Analysis of Variance (PERMANOVA) through the Adonis function in the vegan package with 999 permutations.

The microbiome contingency table was processed using the *Normalization* function from the MetaboAnalystR package in R. Data were median-normalized across samples, log-transformed, and auto-scaled (mean-centered and divided by each species’ standard deviation). These procedures minimized technical variation and better approximated the data’s normal distribution, thereby satisfying the assumptions for parametric tests. The differential abundance between treatments was obtained by a paired t-test applied to the normalized data. Fold Changes were calculated as the ratio between two group means using the data before normalization. The volcano plot summarizing the log_2_ Fold Change and the −log_10_(p-value) was generated by the ggplot package in R.

Raw sequences were submitted to NCBI SRA and can be assessed with BioProject accession number PRJNA1143454.

### Proteomics

In all mouse subjects, a 1 cm segment was cut from the distal colon, where the colitis was targeted, and the spleen was also harvested in *Agr2*^−/−^ mice and WT controls. Both sample types were flash frozen in liquid nitrogen and stored at −80°C. In DSS-injured mice, the distal colon was also collected but preserved with 10% formalin. Protein extraction and digestion were performed by the University of Florida Mass Spectrometry Research and Education Center using established protocols as previously described by Boeno et al^104^. Briefly, colon and spleen tissues were processed with the EasyPep^™^ MS Sample Prep Kit (Thermo Fisher Scientific), quantified by Qubit, and digested using the Rapid-Digestion Trypsin/Lys-C Kit (Promega). Each sample contained 100 μg total protein and was reduced with dithiothreitol, alkylated with iodoacetamide, digested at 70°C for 1 h, and quenched with 0.5% trifluoroacetic acid.

LC-MS/MS was performed on a Q Exactive HF Orbitrap mass spectrometer coupled to an UltiMate 3000 RSLCnano system with an EASY Spray source and a PharmaFluidics 50 cm mPAC^™^ column maintained at 40°C, using the gradient conditions reported previously^104^. MS/MS data were processed in Proteome Discoverer 3.0.1.27 using Sequest^™^ search against Mus musculus, TaxID 10090, with a parent-ion tolerance of 10 ppm and fragment-ion tolerance of 0.020 Da. Carbamidomethylation of cysteine was set as a fixed modification, while methionine oxidation, methionine loss, and N-terminal acetylation were variable. Protein abundance datasets were filtered to remove low-confidence identifications.

The filtered data were uploaded into MetaboAnalyst 6.0 for downstream processing. Missing values were imputed with one-fifth of the minimum detected value for each respective variable. To correct for inter-sample variability, the dataset was median-normalized, log10-transformed, and range-scaled before statistical analysis. Proteomic differential expression was analyzed in R using one-way ANOVA, and proteins with FDR < 0.05 and log_2_FC > 1 were considered significant. Post hoc Tukey’s HSD tests provided pairwise comparisons, and significance groupings were denoted by letters derived using the rstatix package. Effect sizes (ω^2^) were calculated to quantify biological impact.

The mass spectrometry proteomics data have been deposited to the ProteomeXchange Consortium via the PRIDE repository with the dataset identifier PXD070273.

### Transcriptomics

Whole-blood RNA was extracted from terminal frozen blood clots collected on day 8 of the DSS recovery phase using a modified TRIzol LS protocol based on the Genomic Medicine Biorepository protocol for RNA isolation from human peripheral blood^105^. The procedure followed the published workflow for erythrocyte lysis, TRIzol-based acid–phenol extraction, and isopropanol precipitation, with minor modifications to accommodate frozen clots and downstream sequencing requirements. Specifically, frozen clots were homogenized in 1× RBC lysis buffer (NH_4_Cl/KHCO_3_/EDTA) using a bead mill homogenizer, followed by standard TRIzol LS extraction and phase separation. After RNA precipitation and ethanol washing, a secondary ethanol reprecipitation step using 3 M sodium acetate (pH 5.2) and 100% ethanol was included to ensure phenol removal and improve purity. RNA concentration and integrity were verified by Agilent 4200 TapeStation system (Agilent Technologies, Santa Clara, CA, USA).

RNA libraries were prepared by the UF Interdisciplinary Center for Biotechnology Research and sequenced on an Illumina NovaSeq 6000 platform to generate paired-end reads of 150 bp. Raw reads were trimmed and quality-checked using FastQC. Reads were aligned to the Mus musculus reference genome (GRCm39) using the STAR RNA-seq aligner^106^, and gene-level quantification was performed with featureCounts^107^. Low-abundance genes were filtered to retain transcripts with a total count ≥ 30 across all samples. Differential expression analysis was conducted in DESeq2^108^, applying a BH correction (adjusted p < 0.05) to control the false discovery rate.

Raw and processed RNA-seq data can be accessed in the ArrayExpress repository under accession code E-MTAB-16067.

### Functional Enrichment and Pathway Analysis

Functional enrichment was performed using Gene Set Enrichment Analysis (GSEA)^109^ and Ingenuity Pathway Analysis (IPA)^110^. For GSEA, genes or proteins were converted to Entrez IDs with *bitr()* from *org.Mm.eg.db*, ranked by log_2_ fold change, and analyzed using *gseGO()* (*clusterProfiler*, *fgsea*) against Gene Ontology Biological Process (GO:BP) terms (*minGSSize* = 10, *maxGSSize* = 500, *p* < 0.05). Enrichment significance was determined from normalized enrichment scores (NES) and Benjamini-Hochberg adjusted q-values. For IPA (Qiagen), differentially expressed genes and proteins filtered by adjusted p-value and log_2_ fold change were uploaded for core and upstream regulator analyses, with networks ranked by activation z-score and −log(p-value).

## Supplementary Files

This is a list of supplementary files associated with this preprint. Click to download.

- FigureS1.png
- FigureS2.png
- FigureS3.png
- FigureS4.png
- FigureS5.png
- FigureS6.png
- FigureS7.png
- FigureS8.png
- FigureS9.png
- SupplementaryTables.xlsx
- SupplementaryFigureCaptions.docx
